# Cancer Vaccines: Research and Applications

**DOI:** 10.3390/cancers11081041

**Published:** 2019-07-24

**Authors:** Vasso Apostolopoulos

**Affiliations:** Institute for Health and Sport, Victoria University, Melbourne 3030, Australia; vasso.apostolopoulos@vu.edu.au; Tel.: +613-9919-2025

**Keywords:** cancer vaccines, checkpoint markers, immune suppression, tumor microenvironment, tumor associated antigen, immunotherapy, antigen delivery

## Abstract

Designing cancer vaccines has been at the forefront of cancer research for over two-and-a-half decades. In particular, delivery methods used to stimulate effective and long-lasting immune responses have been the major focus. This special issue presents new tumor associated antigens, delivery methods, combination immune therapies, methods of measuring immunity induced following cancer vaccinations, and mechanisms in understanding tumor microenvironments and immunosuppression—all beneficial for the design of improved cancer vaccines.

Cancer vaccines or immunotherapeutics are increasingly recognized as key approaches against cancer. In the last two decades, a number of methods of antigen delivery have been developed, some of which have shown strong anti-tumor immune responses and clinical responses in humans (Figure 1). The combination of immune checkpoints and immune cell therapies has refocused our attention toward improved anti-cancer effects. Different vaccine strategies are presented in this special issue and reviewed by Pender et al. [1]. A tumor’s microenvironment, associated molecules (soluble factors, immunosuppressive markers), immune cell infiltration, and altered extracellular matrix contribute to tumor growth and metastasis [2,3,4,5,6]. As a result, these elements create a microenvironment favorable for tumor progression, and the tumor is able to escape from the host’s immune system. It is important when designing cancer vaccines that there is clear knowledge of a tumor’s microenvironment and immunosuppressive factors, as these will greatly influence the outcome of immune therapeutic approaches [7]. 

Identification of tumor associated antigens is also required when designing cancer vaccines, in order to stimulate the immune system against cancer cells. One example is Mucin 1 (MUC1), which is highly expressed on adenocarcinomas. Numerous vaccines for MUC1 have been designed and tested in preclinical and clinical studies [8,9,10]. Human epidermal growth factor receptor 1 (HER2/neu) has also been a target where anti-HER2/neu antibodies block cancer progression. The paper by Carla de Giovanni et al. shows that co-targeting of HER2/neu and insulin-like growth factor receptor-1 (IGF1R) induces significant antibody responses and delays the onset of spontaneous rhabdomyosarcoma cells [11]. Neoantigens derived from tumor-specific genetic mutations can also be suitable targets in cancer vaccine studies due to their highly immunogenic nature. The immunogenicity of 10 driver mutations, which are commonly expressed in a number of cancers, were tested for their immunogenic ability using peripheral blood mononuclear cells. Six out of 10 peptides stimulated cluster of differentiation 4 (CD4)+ and/or CD8+ T cells [12]. Since neoantigens can be shared by a number of cancer types that are not usually lost due to immune escape, they may be a promising choice to be used as cancer vaccine targets in immunotherapeutic approaches. Another promising antigenic cancer target is the cancer testis sperm protein 17 (Sp17), which is highly expressed by ovarian cancer cells. Sperm protein 17 is not involved in tumor proliferation in vitro but is crucial for its growth in vivo, expressing high levels of signal transducer and activation of transcription 3 (STAT3) and program death ligand 1 (PD-L1) and lower levels of major histocompatibility (MHC) class II on the epithelial ovarian cancer cell line, ID8. Interestingly, the cell population Sp17high/PD-L1+/MHCII- was more resistant to paclitaxel induced cell death in vitro, compared to the Sp17low/PD-L1-/MHCII+ cell population, and was associated with a higher STAT3 expression. Given this information, Sp17 is a suitable target for further cancer vaccine development [13].

In the last 2 decades, a number of vaccine delivery methods have been developed, all with the aim of inducing strong immune responses. An array of methods have shown to be effective in pre-clinical models and some in human clinical trials. Examples of successful antigen delivery methods include adjuvants (Ribi, aluminum hydroxide, Freund’s (although not for human use), and QS21), nanoparticles, virus like particles, the multiple antigen approach, immunostimulatory complexes, viral vectors, DNA delivery methods, cell penetrating peptides, activation of toll-like receptors, chemokine receptors, and dendritic cell receptor targeting [14]. In this special issue, a novel tumor antigen delivery method using endogenous engineered exosomes is presented against human papilloma virus (HPV)-associated neoplasms, with induction of anti-tumor CD8+ T cells [15]. In addition, targeting CD169+ (sialic acid receptor) antigen presenting cells via anti-CD169 antibody-antigen conjugates has also been shown to be effective in stimulating specific T cell responses. In fact, anti-gp100 and Trp2 specific T cells were induced in mice and anti-melanoma-associated antigen recognized by T cells (MART-1) in human leukocyte antigen (HLA)-A2.1 transgenic mice [16]. The SA-4-1BBL ligand for the 4-1BB co-stimulatory receptor of the tumor necrosis factor superfamily has been shown to be an effective anti-cancer approach in a number of tumor models. However, the production of a soluble ligand has hindered the progression of this approach. In this special issue, Garza-Morales et al. show that a chimeric HPV-16 E7 DNA vaccine with SA-4-1BBL was able to induce greater protection against TC-1 cancer cells in mice compared to controls, with strong anti-E7 IFN-gamma secreting T cells [17]. In the early 1990’s, Apostolopoulos et al. targeted MUC1 tumor associated antigens to dendritic cells (in particular, mannose receptor targeting), which induced highly specific immune and clinical responses in animal and in human clinical trials [18,19]. Later, it was shown that the ligand mannan used to target antigens in the mannose receptor resulted in the maturation of dendritic cells and activation via toll-receptor 4 [20,21]. Another approach to increase the maturation of dendritic cells is the use of granulocyte-macrophage colony stimulating factor (GM-CSF) and IL-15 which decreases the prostaglandin E2 pathway and induces an even more immunogenic dendritic cell phenotype by increasing Th1 cells and increasing the survival of ovarian cancer cells in mice [22]. 

In a tumor’s microenvironment, dendritic cells are polarized into immune suppression, which affects T cell activation and supports the differentiation of regulatory T cells leading to tumor growth. As such, incorporating small interfering RNAs and short-hairpin RNAs against immunosuppressive factors within dendritic cells shows promising anti-cancer effects [23]. The combination of short-hairpin RNAs/small interfering RNAs, and dendritic cell based vaccines is also discussed [24]. In addition, the combination of adipose derived stem cells and the E7 cancer antigen specific protein co-injected into mice significantly reduced the endothelial cell marker CD31, the vascular endothelial growth factor, and increased CD4+ T cells, CD8+ T cells, and natural killer cells. Thus, this combination is a promising immunotherapeutic approach for further pre-clinical and human clinical trials [25].

DNA vaccines have been shown to be effective following intramuscular, gene gun, electroporation, or bioinjector delivery methods. Animal-derived hyaluronidase has been shown to be a good enhancer of intramuscular gene electro-transfer in anti-cancer vaccination protocols in mice. A new recombinant hyaluronidase was developed by de Robertis et al., as reported in this special issue, with no toxicity, good plasmid in-take ability, and activation of appropriate immune responses [26]. In addition, oncolytic viruses (natural or genetically modified, in particular, vaccinia virus, reovirus, and myxoma virus), although studied for over 3 decades, have recently emerged as viable immune therapies, with two viruses now approved for cancer treatment in humans. The advantages and benefits of oncolytic viruses are reviewed in this special issue, as well as addressing issues such as cancer specificity, dose, the induction of appropriate immune responses, overcoming pre-existing antibodies from previous exposure to the virus, and safety profiles [27].

Combination immune therapy is becoming popular to treat cancers. In particular, checkpoint markers have been used in combination with chemotherapy and other treatments for improved outcomes. Indeed, the CD200 immune checkpoint inhibitor and tumor lysates in combination enhances anti-glioma T cells and prolonged survival in a canine spontaneous glioma model [28]. Another approach is the use of the IMA950/poly-ICLC peptide vaccine in combination with bevacizumab. An improved response to bevaczumab was noted in glioma patients. However, the potential synergy of concomitant administration should be further tested in future human clinical trials [29]. 

A number of methods for measuring immunity induced by cancer vaccines have been developed, such as bioplex or enzyme linked immunospot (ELISpot) cytokine analysis, cytotoxic T cell activity assays, cytotoxic T cell precursor frequency assays, T cell proliferation, flow cytometry analysis, carboxyfluorescein succinimidyl ester (CFSE) analysis, tetramers, and antibody measurements [30]. In this special issue, Wilson AL et al. elegantly present a non-invasive fluorescent imaging method to directly visualize tumor mass and distribution over the course of tumor progression [31]. Changes were also noted in CD8+ T cells, regulatory T cells, dendritic cells, and myeloid derived suppressor cells over time [31], thereby showing a new non-invasive approach for the monitoring of cancer vaccine candidates.

In the last 2 decades, vaccines for cancer have shown promise in pre-clinical and human clinical trials. Identification of tumor associated antigens, antigen delivery methods, induction of immune responses, methods of measuring immunity, and, more recently, tumor microenvironments and immunosuppressive factors have all contributed to improved cancer outcomes in patients (Figure 1). Different methods are being tested in human clinical trials and we await their outcomes to determine which method or combination of methods will prove useful in eradicating disease. More recently, nutrition [32,33] and exercise [18,33,34,35] have also shown promise as a means of boosting the immune system, and cancer vaccines in combination with exercise or nutrition may further improve clinical outcomes. Obesity and chronic inflammation [36] have also contributed to the cancer endemic, and understanding the complex immunological and inflammatory network in obesity and chronic inflammatory conditions is required to design optimal vaccine immune therapeutic strategies for cancer. 

## Figures and Tables

**Figure 1:**
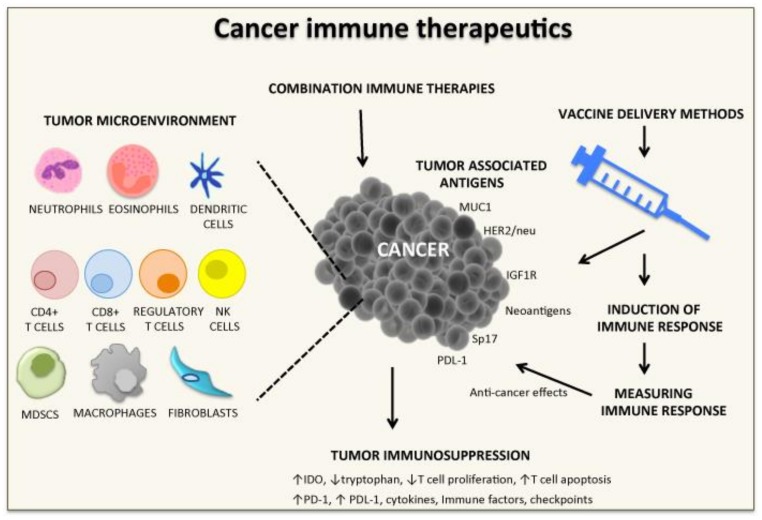
Strategies for cancer vaccine development. Strategies presented in this special issue on Cancer Vaccines: Research and Applications. CD, cluster of differentiation; HER1/neu, Human Epidermal Growth Factor Receptor 2; IDO, Indoleamine-2,3-dioxygenase; IGF1R, insulin-like growth factor receptor-1; MDSCS, Myeloid-derived suppressor cells; MUC1, Mucin 1; NK, natural killer; PD-1, programmed death-1; PDL-1, program death ligand 1; Sp17, cancer testis sperm protein 17.
